# Early Functional Impairment in Experimental Glaucoma Is Accompanied by Disruption of the GABAergic System and Inceptive Neuroinflammation

**DOI:** 10.3390/ijms22147581

**Published:** 2021-07-15

**Authors:** Oliver W. Gramlich, Cheyanne R. Godwin, David Wadkins, Benjamin W. Elwood, Markus H. Kuehn

**Affiliations:** 1 Department of Ophthalmology and Visual Sciences, The University of Iowa, Iowa City, IA 52242, USA; cheyanne-godwin@uiowa.edu (C.R.G.); david-wadkins@uiowa.edu (D.W.); benjamin-elwood@uiowa.edu (B.W.E.); markus-kuehn@uiowa.edu (M.H.K.); 2 VA Center for the Prevention and Treatment of Visual Loss, Iowa City VA Health Care System, Iowa City, IA 52246, USA; 3 Department of Neuroscience and Pharmacology, The University of Iowa, Iowa City, IA 52242, USA

**Keywords:** glaucoma, myocilin, pattern electroretinography, γ-aminobutyric acid (GABA), neuroinflammation, retinal ganglion cell loss

## Abstract

Glaucoma is a leading cause of irreversible blindness worldwide, and increased intraocular pressure (IOP) is a major risk factor. We aimed to determine if early functional and molecular differences in the glaucomatous retina manifest before significant retinal ganglion cell (RGC) loss is apparent. Adenoviral vectors expressing a pathogenic form of myocilin (Ad5.MYOC) were used to induce IOP elevation in C57BL/6 mice. IOP and pattern electroretinograms (pERG) were recorded, and retinas were prepared for RNA sequencing, immunohistochemistry, or to determine RGC loss. Ocular injection of Ad5.MYOC leads to reliable IOP elevation, resulting in significant loss of RGC after nine weeks. A significant decrease in the pERG amplitude was evident in eyes three weeks after IOP elevation. Retinal gene expression analysis revealed increased expression for 291 genes related to complement cascade, inflammation, and antigen presentation in hypertensive eyes. Decreased expression was found for 378 genes associated with the γ-aminobutyric acid (GABA)ergic and glutamatergic systems and axon guidance. These data suggest that early functional changes in RGC might be due to reduced GABA_A_ receptor signaling and neuroinflammation that precedes RGC loss in this glaucoma model. These initial changes may offer new targets for early detection of glaucoma and the development of new interventions.

## 1. Introduction

Glaucoma is a neurodegenerative eye disorder, which is characterized by the irreversible degeneration of retinal ganglion cells (RGC), leading to visual impairment and blindness [1,2]. In addition to age, elevated intraocular pressure (IOP) is a major risk factor for glaucoma [3,4,5]. The multifactorial pathobiology of elevated IOP and RGC death is complex and involves changes in connective tissue composition and biomechanical support, blood flow and vascular insufficiency [6,7], metabolic and oxidative stress [8,9,10], neuroinflammatory and immune mechanisms [11,12], but also genetic factors [13], such as mutations in the myocilin (MYOC) gene [14].

McDowell et al. first described a viral vector-inducible glaucoma model in mice on the background of the human MYOC mutation [15]. The authors describe that the administration of an adenoviral 5 (Ad5) vector expressing MYOC (Ad5.MYOC) with a pathogenic mutation into the posterior chamber resulted in IOP elevation with moderate degeneration of the optic nerve. Whilst the pathogenesis of MYOC-induced IOP elevation is not completely understood, McDowell et al. hypothesized that mutated MYOC is misfolded and causes endoplasmic reticulum and cellular stress in trabecular meshwork (TM) cells. In turn, this leads to TM dysfunction and subsequent IOP elevation. We recently used this model to demonstrate a contribution of lymphocytes to progressive RGC loss in advanced glaucoma [16]. In this study, our goal was to determine the magnitude of RGC degeneration after three and nine weeks of chronic IOP elevation. Our second objective is to determine pattern electroretinography (pERG) abnormalities and correlate these with alterations in gene expression occurring as early as three weeks after induced ocular hypertension.

Previous studies using the bead occlusion model, in which ocular hypertension is induced by the injection of microbeads into the anterior chamber, demonstrate altered RGC responses to light, which is determined ex vivo by multielectrode array recording prior to RGC loss [17,18,19]. These studies further suggest that not only RGC but also the inner retinal circuit is functionally impaired by IOP elevation. There is increasing evidence that synaptic integrity in the inner plexiform layer is affected in glaucoma [20,21], which might be linked to the disruption of the γ-aminobutyric acid (GABA)-ergic and glutamatergic systems [22,23] and could explain the functional impact seen in electrophysiological responses. Furthermore, excessive mechanical stress occurring from IOP elevation triggers stretch and swelling-induced adenosine triphosphate release in the retina. This IOP-related mechanosensitive mechanism(s) includes metabolic stress in Müller cells and subsequently in RGCs, Ca^2+^-dependent dendritic and axonal remodeling, and inflammatory responses [24]. 

Our findings indicate pERG deficits as early as three weeks after Ad5.MYOC injection. At this early timepoint, no significant RGC loss could be determined, but the functional changes are accompanied by changes in gene transcription indicating disturbances in GABA_A_ receptor signaling and GABA recycling, as well as activation of the complement cascade and inceptive neuroinflammation. These findings provide a molecular basis for the observed functional deficits in glaucoma and further underscore that neuroinflammation is an early event in glaucomatous neurodegeneration [21,25,26,27,28]. Additionally, we could determine a significant RGC loss in eyes after nine weeks of chronic IOP elevation, further demonstrating that RGC loss in this model is progressive and requires several weeks to become patent.

## 2. Results

### 2.1. Intraocular Injection of Ad5 Viral Vector Expressing Myocilin Y437H Results in Chronic IOP Elevation and RGC Loss

**Figure 1:**
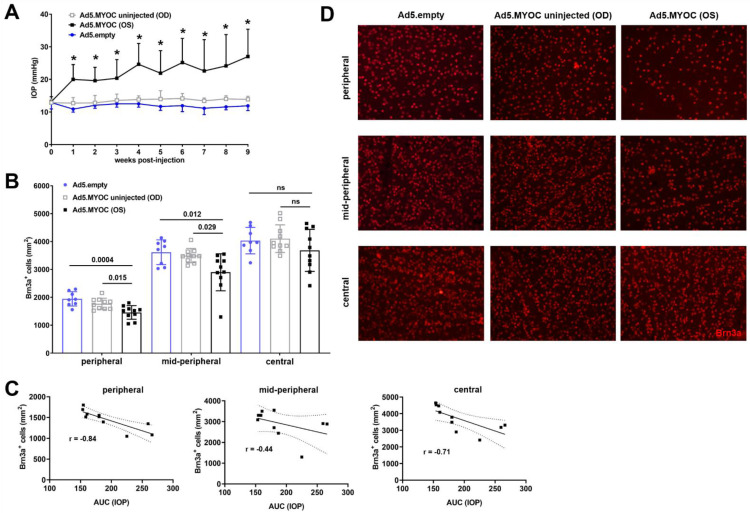
IOP and RGC density in Ad5.MYOC injected eyes. (**A**) Chronic IOP elevation is evident as early as one week after Ad5.MYOC vector injection into the anterior chamber of left eyes (Ad5.MYOC (OS), black squares) when compared to either normotensive contralateral eyes (Ad5.MYOC uninjected (OD), gray squares) or eyes having received Ad5.empty vector (Ad5.empty, blue circles; * *p* ˂ 0.001). (**B**) Loss of Brn3a^+^ RGC in hypertensive eyes nine weeks after intraocular Ad5.MYOC injection when compared to their contralateral uninjected eyes or to RGC density in Ad5.empty vector-injected eyes. Data in A and B are given as mean ± SD. Every point reflects counts from an individual eye. ns = not significant *p* > 0.05. (**C**) Regression analysis of RGC density in Ad5.MYOC injected eyes and Area Under Curve (AUC) IOP values demonstrate strong correlation, especially when using peripheral RGC data. (**D**) Representative images of Brn3a immunostaining of retinal wholemounts display reduced RGC density in the peripheral and mid-peripheral areas after IOP elevation (far right column).

Following intraocular Ad5.MYOC administration, Ad5-desensitized C57BL/6 mice developed significant IOP elevation within one week, and IOP remained chronically elevated at an average of 22.8 ± 2.6 mmHg throughout the time course of nine weeks. The IOP of uninjected contralateral eyes remained at baseline values of about 13.5 ± 0.5 mmHg (Figure 1A). To rule out that intraocular injection of viral vectors per se causes IOP elevation or RGC loss, another cohort of mouse eyes (*n* = 10) were injected with an empty construct of the Ad5 vector (Ad5.empty). Throughout a time course of 9 weeks after ocular delivery, no changes from baseline IOP were noted in these Ad5.empty injected eyes (average IOP: 11.9 ± 0.61 mmHg). 

Nine weeks after Ad5.MYOC injection, analysis of retinal wholemounts indicated a prominent loss of RGC in eyes with ocular hypertension. RGC loss in hypertensive eyes is statistically most significant in the peripheral area of retinas (1455 ± 242 Brn3a^+^ cells/mm^2^) when compared to RGC density in both their uninjected contralateral eyes (1768 ± 199 Brn3a^+^ cells/mm^2^, *p* = 0.015) and to Ad5.empty vector-injected control eyes (1945 ± 256 Brn3a^+^ cells/mm^2^, *p* = 0.0004). RCG reduction in glaucomatous eyes is also evident in the mid-peripheral region (2898 ± 664 Brn3a^+^ cells/mm^2^) when compared to the contralateral eyes (3496 ± 258 Brn3a^+^ cells/mm^2^, *p* = 0.029) or control eyes injected with the Ad5.empty construct (3618 ± 445 Brn3a^+^ cells/mm^2^, *p* = 0.012). In the central retina, RGC loss was observed in some eyes, but statistical significance was not reached (Figure 1B,D). The density of Brn3a^+^ RGC in Ad5.empty injected eyes was similar to that of contralateral control eyes in the peripheral (Ad5.empty: 1945 ± 256 Brn3a^+^ cells/mm^2^ vs. uninjected: 2014 ± 271 Brn3a^+^ cells/mm^2^, *p* = 0.62), mid-peripheral (Ad5.empty: 3618 ± 445 Brn3a^+^ cells/mm^2^ vs. uninjected: 3749 ± 248 Brn3a^+^ cells/mm^2^; *p* = 0.50), and central areas (Ad5.empty: 4034 ± 474 Brn3a^+^ cells/mm^2^ vs. uninjected: 4236 ± 213 Brn3a^+^ cells/mm^2^; *p* = 0.31, *t*-test values were corrected for multiple comparison). Regression analysis of glaucomatous eyes reveals a strong correlation between IOP data and RGC density in the peripheral and central retina, whereas higher Area Under Curve (AUC) IOP relates to the highest RGC loss (Figure 1C). 

These data strongly indicate that in Ad5-desensitized C57BL/6 mice, intraocular administration of the Ad5.MYOC viral vector is safe for the eye and that observed IOP changes in injected eyes are due to the effects of the transgene. In our hands, intraocular injection of Ad5.MYOC injection is an effective and highly reproducible method to induce chronic ocular hypertension with subsequent RGC loss.

### 2.2. Slow Progressive RGC Degeneration after Ad5.MYOC Induced IOP Elevation

**Figure 2:**
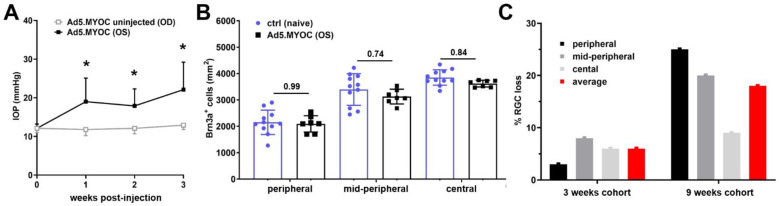
IOP and RCG density three weeks after induction of ocular hypertension. (**A**) The IOP profile indicates substantial induction of ocular hypertension using intraocular Ad5.MYOC injection when compared to their contralateral uninjected eyes. * *p* < 0.05 (**B**) Analysis of RGC density at that early time point indicates no significant loss in hypertensive eyes when compared to age-matched controls. Every point reflects counts from an individual eye. (**C**) Analysis of the percentage RGC loss after three and nine weeks of OHT indicates a slow progressive RGC loss, which is most prominent in the peripheral part of the retina.

To examine functional and molecular changes occurring early in Ad5.MYOC induced ocular hypertension, retinas of a second cohort of mice were harvested three weeks after Ad5.MYOC injection to determine RGC loss and for RNA sequencing. In accordance with our previous results, intraocular Ad5.MYOC administration led to chronic IOP elevation within one week after injection with an average of 19.7 ± 6.2 mmHg, while uninjected contralateral eyes maintained a mean IOP of 12.3 ± 1.4 mmHg (Figure 2A), and normal IOP was observed in naive control mice. Analysis of the RGC density between naive eyes and eyes exposed to ocular hypertension for three weeks does not indicate significant differences in the periphery (Ad5.MYOC: 2093 ± 311 Brn3a^+^ cells/mm^2^ vs. naïve controls: 2153 ± 461 Brn3a^+^ cells/mm^2^, *p* = 0.99), mid-periphery (Ad5.MYOC: 3128 ± 279 Brn3a^+^ cells/mm^2^ vs. naïve controls: 3396 ± 599 Brn3a^+^ cells/mm^2^, *p* = 0.74) and central area of the retina (Ad5.MYOC: 3621 ± 126 Brn3a^+^ cells/mm^2^ vs. naïve controls: 3851 ± 292 Brn3a^+^ cells/mm^2^, *p* = 0.84, Figure 2B). These findings demonstrate that retinal damage after 3 weeks of ocular hypertension is extremely light and, in concert with those obtained from eyes after nine weeks of elevated IOP, highlight the progressive nature of RGC loss in this disease model (Figure 2C).

### 2.3. IOP Elevation Causes Rapid Retinal Expression of Inflammation Markers and Downregulation of GABA_A_ Receptor Subunits and Glutamine Synthetase

**Figure 3:**
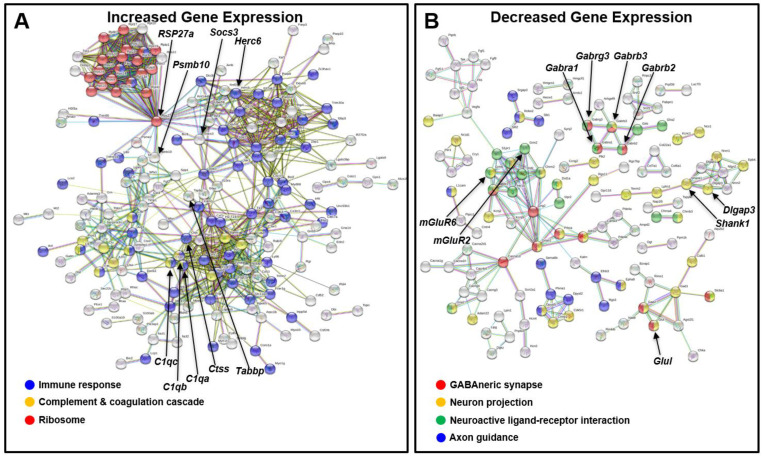
Retinal gene expression cluster analysis of hypertensive eyes using GO and KEGG pathway analysis. (**A**) Increased gene expression in glaucomatous retina early after induced pressure elevation is related to immune response, activation of the complement system, and increased expression of ribosomal genes related to the ubiquitin–proteasome system. (**B**) Decreased expression is found for genes encoding GABA_A_ receptor subunits involving ligand–receptor interaction. Two additional clusters are found for the decreased expression of genes associated with axonal guidance and neuron projection.

Retinal gene expression analysis from eyes after three weeks of increased IOP revealed a significant increase in expression for 291 genes, notably those encoding stress-related proteins such as ɣ-crystallin B (control: 214 ± 96 FPKM vs. Ad5.MYOC: 830 ± 435 FPKM, *p* = 0.033), ɣ-crystallin C (control: 176 ± 65 FPKM vs. Ad5.MYOC: 699 ± 399 FPKM, *p* = 0.041) and also GFAP (control: 986 ± 376 FPKM vs. Ad5.MYOC: 2445 ± 626 FPKM, *p* = 0.007). Increased gene expression was also evident in retinas of hypertensive eyes for complement proteins including *C1q*, *C4,* and the C1-inhibitor *SerpinG1*. The initiation of the complement cascade is accompanied by increased expression of the microglia/macrophages associated marker *Cx3cr1* and of genes linked to antigen processing and presentation, in particular *Ctss*, *Tapbp*, and *H2-t23* (Table 1). Cluster analysis confirmed that the upregulated genes are linked to the complement cascade but also to antigen processing and presentation, immune cells, and cytokine–cytokine receptor signaling (Figure 3A).

Changes in gene expression encoding ribosomal subunits were also observed (Table 1) that are linked to immune response-related genes via *Rps27a* (control: 3821 ± 118 FPKM vs. Ad5.MYOC: 4650 ± 348 FPKM, *p* = 0.004), which is a component of the 40S subunit. *Rps27a* is also known as Ubiquitin B and is one of four genes encoding ubiquitin. The primary function of ubiquitin is to clear abnormal and improperly folded proteins by targeting them for degradation by the 26S proteasome. No differences were observed in the expression of genes indicative of ischemic events. Decreased expression was found for 378 genes, most striking for glutamine synthetase (*Glul*, control: 33,233 ± 4840 FPKM vs. Ad5.MYOC: 47,282 ± 621 FPKM, *p* = 0.001). Furthermore, the expression of genes related to the GABAergic system and genes associated with neuron projection and axonal guidance were found to be significantly decreased (Figure 3B). These include genes with functions related to GABA_A_ receptor and GABA neurotransmitter recycling, genes required for cholinergic and glutamatergic synapse formation, and axonal guidance (Table 1).

Taken together, our gene expression data point to an inceptive neuroinflammation early after IOP elevation with activation of the complement cascade and an increase of cellular immune cell function. This early immune response is accompanied by decreased expression of important components of signal transduction in the neuro-retinal circuit.

### 2.4. Hypertensive Eyes Display Retinal C1q Accumulation Accompanied by Reduced GABA_A_ Receptor and PSD95 Immunolabeling

**Figure 4:**
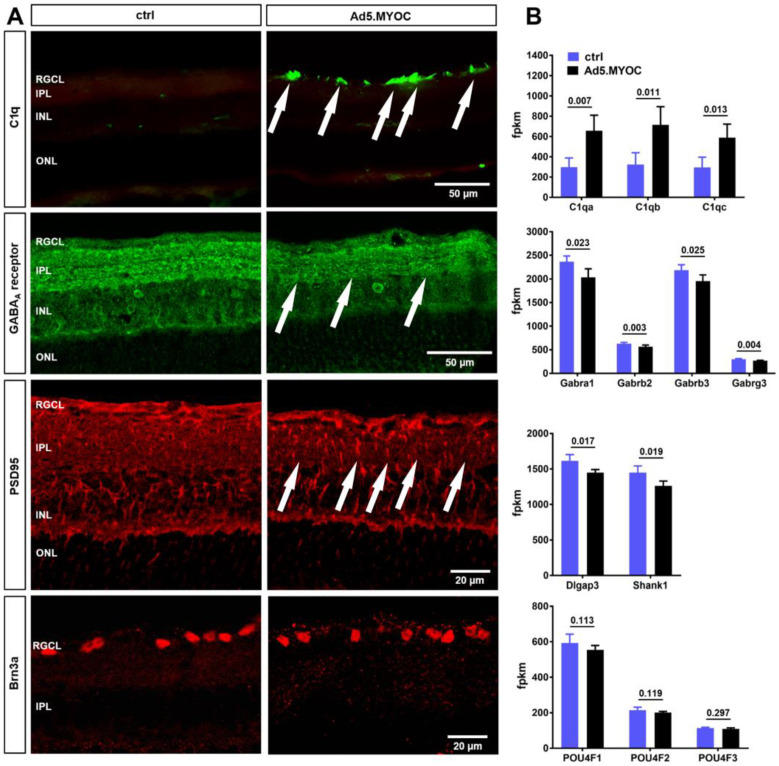
C1q accumulation and altered synaptic labeling in glaucomatous retinas. (**A**) Retinas of hypertensive eyes (Ad5.MYOC) display obvious accumulation of complement component C1 in the retinal ganglion cell layer (RGCL, arrows) when compared to naïve control retinas (ctrl). Decreased immunolabeling is evident for GABA_A_ receptor and PDS95, indicating synaptic loss in the inner plexiform layer (IPL, arrows). Decrease in Brn3a positive cells was not noticed after 3 weeks of chronic IOP elevation (INL = inner nuclear layer, ONL = outer nuclear layer). (**B**) Changes in immunohistochemical labeling are in accordance with expression changes of corresponding genes. Values of fragments per kilobase of transcript per million mapped reads (FPKM) are given as mean ± SD and *p*-values were calculated using Student’s *t*-test.

To further confirm the increased expression of inflammatory markers in the retina after Ad5.MYOC injection, we conducted immunostaining for C1q in retinal cross-sections of eyes after three weeks of elevated IOP. Abundant C1q deposition was observed in the retinal nerve fiber layer and the RGC layer of hypertensive eyes, but punctuated C1q labeling was also evident in the inner plexiform layer (IPL, Figure 4A). This increase in C1q labeling is concordant with increased expression levels of the genes *C1qa*, *C1qb,* and *C1qc* (Figure 4B). Conversely, immunolabeling for the GABA_A_ receptor revealed disruption of the three specific GABA_A_ receptor positive strata that are normally present in the IPL and are clearly visible in our controls. Overall, immunolabeling of the GABA_A_ receptor was notably weaker in the IPL. A decrease of GABA_A_ receptor positive neurons was also observed in the inner nuclear layer of retinas from hypertensive eyes when compared to controls (Figure 4A). This decrease is mirrored by reduced expression levels of genes related to the GABA_A_ pathway including *Gabra1*, *Gabrb2* and *3*, and *Gabrg3* (Figure 4B).

Analogous to GABA_A_ receptor patterns, PSD95 immunohistochemistry revealed decreased labeling of this synaptic marker in the IPL of retinas exposed to ocular hypertension (Figure 4A). A corresponding decrease in SH3 and multiple ankyrin repeat domains protein 1 (*Shank1*) and Disks large-associated protein 3 (*Dlgap3*) genes in the Postsynaptic density protein 95 (PSD95) complex was also seen (Figure 4B). PSD95 labeling is typically pronounced in the outer plexiform layer and is also present there in our model. Labeling intensities do not differ between the glaucomatous group and controls in this region, indicating that postsynaptic modification occurred specifically in RGC. 

Our finding that a significant RGC loss had not yet occurred after three weeks of ocular hypertension is further supported by the observation that expression levels of genes of the POU4 family, which are related to RGC survival and health, are similar in eyes after Ad5.MYOC administration and controls (Figure 4A,B). Thus, other observed changes in transcript levels are a result of modulation of gene expression rather than loss of RGC.

These results confirm our previous findings of increased complement activation but also demonstrate decreased GABA_A_ receptor and PSD95 gene expression and immunolabeling after Ad5.MYOC-mediated IOP elevation. 

### 2.5. IOP Elevation Causes Decline in Pattern ERG Amplitudes

**Figure 5:**
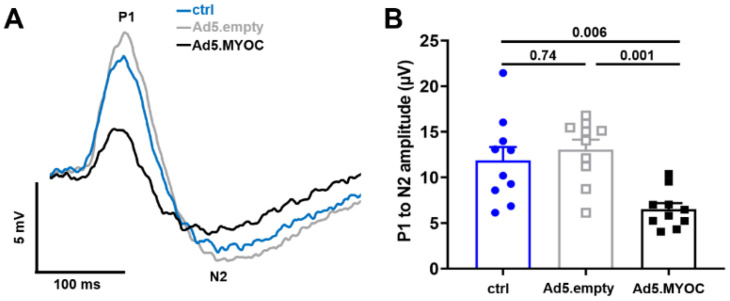
Pattern electroretinography (pERG) three weeks after IOP elevation. (**A**) Overlay of averaged traces demonstrating functional impairment in the 1 Hz pERG of hypertensive eyes (Ad5.MYOC, black squares) while age-matched naïve controls (ctrl, blue circles) and mice having received the empty Ad5 construct (Ad5.empty, gray squares) show normal pERG waveforms. (**B**) The P1 to N2 amplitude is significantly decreased when compared to naïve or Ad5.empty eyes. Scatter plots reflect data from individual eyes per group.

We further wanted to investigate whether the molecular changes in the GABAergic system and in synaptic plasticity that occur early after IOP elevation result in corresponding functional deficits. In addition, we wanted to rule out that functional changes are a consequence of ocular Ad5.vector injections per se. Therefore, we compared pERG data obtained from a third cohort of mice (including naïve controls) three weeks after Ad5.MYOC-induced hypertension and eyes injected with Ad5.empty vector. Similar to our previous findings, only eyes having received Ad5.MYOC display significant IOP elevation with an average of 17.6 ± 1 mmHg when compared to a mean IOP of 11.7 ± 1.5 mmHg (*p* < 0.001) in naïve eyes and to 11.5 ± 1.2 mmHg (*p* < 0.001) in Ad5.empty-injected eyes. Eyes with Ad5.MYOC-induced IOP elevation displayed a decrease of approximately 1/3 of their P1 to N2 amplitude (Figure 5A). This reduction is significant in comparison to the amplitudes of Ad5.empty-injected eyes (Ad5.MYOC: 14.1 ± 4 µV vs. Ad5.empty: 23.8 ± 8.5 µV, *p* = 0.048) as well as to those of age-matched naive controls (control: 25.9 ± 6.7 µV, *p* = 0.004; Figure 5B). Shortening or prolonged latencies have not been detected in any hypertensive eyes after Ad5.MYOC injection.

These data demonstrate that the administration of an Ad5.empty viral construct does not provoke functional changes in the retina. However, ocular administration of an Ad5.vector expressing MYOC with subsequent IOP elevation leads to significant functional impairment that precedes RGC loss.

## 3. Discussion

### 3.1. Intraocular Injection of Ad5.MYOC Leads to Ocular Hypertension, Functional Decline, and Eventual RGC Loss

In this study, we employed intraocular injection of adenovirus 5 vector expressing a pathogenic form of human Myocilin, Ad5.MYOC^Y437H^, to induce chronic ocular hypertension in C57BL/6 mice to investigate early events. In our hands, the increase in IOP, as well as the profile and magnitude of IOP elevation are comparable to those described earlier [15,16]. The degeneration of RGC is not associated with the intraocular administration of the viral vector itself, since the injection of empty Ad5 viral vector into the anterior chamber causes neither IOP elevation nor RGC loss. Thus, Ad5.MYOC-mediated IOP elevation is a simple, safe, and reliable approach to induce glaucomatous changes in mice.

Here, we report that functional impairment of the retina as determined by pERG is present shortly after IOP elevation and that these changes precede significant RGC degeneration. RGC dysfunction is evident through significantly decreased amplitude in hypertensive eyes when compared to both contralateral uninjected eyes and to eyes of the naïve control group, which were observed prior to apparent RGC loss. This observation is in accordance with clinical glaucoma studies [29,30,31] and preclinical models of glaucoma [32,33,34] in which pERG was used to detect RGC (dys-)function. 

### 3.2. Functional Impairment in Hypertensive Eyes Is Accompanied by Synaptic Degeneration, Altered Glutamine Synthase, and Decreased GABA_A_-Receptor Expression

Our gene expression data may help to explain the origin of the functional impairment early after IOP elevation. In general, gene expression changes observed in our model are similar to those reported elsewhere and include the increased expression for GFAP as a marker for gliosis [35], stress proteins [36], and the downregulation of the glutamate synthetase [37]. We also found a significant increase in the expression of classical complement cascade proteins, especially for C1q. Activation of the complement system has been previously shown in glaucoma animal models [38,39,40] and human glaucomatous retinas [41,42]. Importantly, no differences in gene expression levels between glaucomatous and controls eyes were noticed for members of the Pou4 family (including *Pou4F1*-encoding Brn3a), suggesting that RGC are still relatively healthy 3 weeks after chronic IOP elevation. This finding was further confirmed by Brn3a immunolabeling in retinal wholemounts for the quantitation of RGC loss, demonstrating undiminished RGC density at this early time point. Thus, it is reasonable to conclude that the changes in retinal gene expression and electrophysiology reported here precede RGC loss in hypertensive eyes. 

The observed decrease in the expression of genes related to GABA_A_ receptor signaling, accompanied by impairment of GABA recycling due to decreased glutamine synthase, and the downregulation of genes related to cholinergic and glutamatergic synapses are consistent with the observed functional impairment. In addition to RGC, GABA_A_ receptors are expressed on horizontal, bipolar, and amacrine cells [43] and play an extraordinarily complex role in signal transduction [44,45]. Depending on the nature of GABA_A_ receptor positive neurons and the amount of GABA present, the GABAergic system can act either inhibitory or excitatory. Our immunohistochemistry shows that ocular hypertension leads to reduced GABA_A_ receptor subunit expression as well as to decreased GABA_A_ receptor immunolabeling in the IPL and in INL neurons. One explanation for these findings is that an early retinal response to elevated IOP impairs GABA_A_-mediated signal transduction between INL neurons and RGC, which would lead to observed pERG deficits. 

A decrease in glutamine synthase levels has also been observed in a variety of experimental glaucoma models [46,47]. Reduced levels of this enzyme could lead to increased glutamate levels and potentially to a microenvironment of glutamate toxicity [48,49], but more directly, they will cause the depletion of available intracellular glutamine, which is an important excitatory neurotransmitter [50,51]. It has been shown that the inhibition of glutamine synthase suppresses the b-wave response and blocks glutamatergic neurotransmission [52], which may further contribute to the decrease in amplitudes seen in our glaucoma model. Finally, it is also plausible that the reduction in metabotropic glutamate receptors (mGluR) 2 and 6 (Table 1) in the glaucomatous retinas has an important role in impaired electrical input into RGC. mGluR6 is believed to be expressed on bipolar cells, and reduced mGluR6 expression would directly alter bipolar cell function [53] and RGCs only indirectly i.e., by propagation of altered bipolar cell signaling. This notion of impaired outer retinal function is further supported by other studies in which the reduction of a- and b-wave amplitudes after induction of ocular hypertension as well as impaired bipolar cell function in the context of glaucoma have been reported [54,55,56]. 

While reduction in glutamatergic signaling is likely to result in decreased synaptic transmission, the decline in transcript levels for *Shank1* and *Dlgap3* (alias SAP90/PSD-95-Associated Protein 3), which are both components of the PSD95 synaptic complex [57,58], is indicative of physical synaptic uncoupling. In our glaucoma model, PSD95 immunolabeling is reduced in the RGC layer and IPL of hypertensive eyes in a pattern similar to that observed in the DBA/2J glaucoma mouse model where a decline in PSD95 indicates synaptic loss [21]. Furthermore, decreased PSD95 immunolabeling accompanied by a reduction in RGC function has also been reported in mice after short-term IOP elevation [59].

Taken together, the selective downregulation of GABA_A_ receptors, decreased glutamine synthase levels, and loss of glutamatergic synapses are likely responsible for the pERG deficits. However, it remains to be determined whether these processes act in concert with one another or if one of them is primarily responsible for the change in RGC function. 

### 3.3. Inceptive Neuroinflammation Features C1q Deposition and Antigen Presentation

As we have previously shown [38], C1q is predominantly located in the nerve fiber layer and RGC layer of the glaucomatous retina. Here, a similar C1q staining pattern, as well as some punctuated C1q immunolabeling in the IPL of retinas exposed to ocular hypertension, was observed. It is possible that C1q deposition is directly involved in synaptic removal, which is a suggested mechanism of synaptic stripping [60], but C1q deposition is certainly indicative of neuroinflammation. It is intriguing that we also detected a profound increase of *Ctss* expression at this early stage of glaucomatous damage. *Ctss* encodes Cathepsin S, which has a critical role in loading antigens to the MHCII complex for the presentation of B-cells, microglia, and macrophages, as well as dendritic cells [61,62]. A recent pathway analysis of GWAS data revealed an association of several histocompatibility (MHC) subtypes along with *Ctss* and heat shock protein (HSP) 70, HSPA1B, with primary open angle glaucoma [63]. In our study, we detected increased retinal expression of H2-T23, a mouse MHCI complex, as well as Tap binding protein (*Tapbp*), which is crucial for the immune response of HSP-involved antigen cross-presentation [64,65]. 

Furthermore, the increased expression of *Rps27a*, alias Ubiquitin B, and its association with the ribosomal 40S subunit, as well as with genes of the proteasomal degradation pathway (*Psmb10* [66], *Socs3* [67], *Herc6* [68]), is indicative that the ubiquitin–proteasome system is activated in the retina of hypertensive eyes. Studies report activation of the ubiquitin–proteasome system in retinal degeneration, including glaucoma, as a result of increased occurrence of misfolded proteins in response to oxidative stress and NF-κB signaling [68,69]. In its role as an immunoproteasome, *Psmb10* is also involved in MHCI antigen presentation [66]. Together, these findings are indicative of MHCI complex-mediated presentation of HSP-derived peptides to cytotoxic CD8 T-cells and are similar to those obtained in our earlier study describing gene expression changes in canine retinas with advanced glaucoma [70,71,72].

HSPs and HSP autoimmunity appear to play a critical role in the pathophysiology of glaucoma [73,74], and it has been demonstrated that ocular delivery or systemic immunization of rodents with HSPs elicits RGC loss [74,75,76]. A more recent study reveals that IOP elevation causes an HSP-specific T-cell response in mice and that RGC loss can be induced by transferring HSP-specific T-cells from glaucomatous donor mice into healthy recipient mice [77]. While it is currently unclear how the immune response to HSP in glaucoma is initiated, there is mounting evidence that it is contributing to RGC loss [78]. Our findings in this study indicate that an HSP-induced immune response might have its origin in the retina and that enhanced antigen presentation occurs very early, prior to significant RGC loss. 

In summary, Ad5.MYOC-induced IOP elevation in MYOC desensitized mice leads to slow progressive RGC loss that is accompanied by a declined pERG amplitude. The functional changes seem to be linked to the decreased expression of genes related to neurotransmitter recycling and receptor expression in the GABAergic and glutamatergic systems and to inceptive neuroinflammatory processes. Of particular interest for further studies is our observation of HSP-related antigen presentation, which may point to a potential mechanism for the development of an autoimmune response in glaucoma.

## 4. Materials and Methods

### 4.1. Animals

All animal experiments have been approved by the University of Iowa and the Iowa City VA IACUC and were conducted in accordance with the ARVO Statement for the Use of Animals in Ophthalmic and Vision Research. C57BL/6/J (B6) mouse breeder pairs were purchased from The Jackson Laboratory (Bar Harbor, ME, USA) and housed at the University of Iowa on a 12 h light–dark cycle (6 a.m. to 6 p.m.) with food ad libitum. Equal numbers of both sexes were used in our experiments, and all animals were euthanized by CO_2_ inhalation followed by cervical dislocation.

### 4.2. Myocilin Vector-Induced Glaucoma Model

An adenoviral vector expressing the pathogenic Y437H form of human Myocilin, Ad5RSVmyocillin^Tyr437His^Flag (Ad5.MYOC) was used to induce IOP elevation [15,16]. Control eyes received an empty (null) vector variant of Ad5CMV (Ad5.empty). Both vector constructs were obtained from the Vector Core at University of Iowa, IA, USA. Newborn pups (P2–P5) were desensitized to Ad5 viruses by a subcutaneous injection of 3 μL of the Ad5.MYOC with a titer of 3 × 10^6^ pfu into the lower back. At two months of age, desensitized mice were anesthetized using 4% isoflurane (Baxter, Deerfield, IL, USA) for induction and 2.5% isoflurane for maintenance at a flow rate of 1 L O_2_/min. 1% tropicamide (Sandoz, Princeton, NJ, USA) and 0.5% proparacaine hydrochloride (Bausch + Lomb, Bridgewater, NJ, USA) eye drops were administered for dilation and local anesthesia, respectively. A 10 μL Hamilton syringe with a 30-gauge needle was inserted into a dorsolateral location of the eye posterior to the limbus, and 3 μL of the Ad5.MYOC with a titer of 9 × 10^7^ pfu were injected over a time of 30 s. Prior to injection, aqueous humor was allowed to escape, so that the injection of an additional volume did not cause undue IOP elevation. After injection, the needle was kept in place for another 30 s to avoid washout. Pulsation and coloration of blood vessels were monitored through a surgical microscope (Omano, Roanoke, VA, USA) to ensure that ischemic damage did not occur. In the first in vivo study, a group of mice (*n* = 10) received a unilateral intraocular injection to determine IOP elevation and RGC loss nine weeks after Ad5.MYOC injection. A control group (*n* = 8) received injections of 3 μL Ad5.empty vector. 

In the second cohort, which was designed to evaluate changes prior to RGC loss, experimental mice received a bilateral Ad5.MYOC injection, and tissue was harvested three weeks thereafter for RNA sequencing (*n* = 8), RGC quantitation (*n* = 10), and immunohistochemistry (*n* = 7). Data were compared to those derived from age-matched naïve animals (*n* = 5 for RNA sequencing, *n* = 8 for RGC quantitation, and *n* = 8 for immunohistochemistry). 

Finally, pERG responses were examined in a third cohort of mice (*n* = 5/group) three weeks after bilateral Ad5.MYOC or Ad5.empty injections, and recordings were compared to a naïve control group. Animals were euthanized after pERG recordings, and eyes of both groups were subsequently used for immunohistochemistry.

### 4.3. Tonometry

An iCare Tonolab rebound tonometer (iCare, Espoo, Finland) was used to monitor the IOP in experimental and control eyes, as previously described [79]. Briefly, mice were anesthetized with isoflurane as described above and the average IOP of 5 consecutive measurements was calculated. IOP was measured between 10 a.m. and 1 p.m. by an investigator masked to the animals’ status. Measurements were obtained prior to ocular viral vector administration and then weekly throughout the study.

### 4.4. Pattern ERG Recording

Mice were anesthetized with an intraperitoneal injection of ketamine (30 mg/kg, Mylan, Canonsburg, PA, USA), xylazine (5 mg/kg, Akorn Inc., Lake Forest, IL, USA), and acepromazine (2.3 mg/kg, Diamondback drugs, Scottsdale, AZ, USA). A drop of GenTeal gel (Alcon Laboratories, Fort Worth, TX, USA) was placed on the corneal surface to maintain corneal integrity, after pupil dilation using 1% tropicamide. pERG responses were recorded using alternating, reversing, black and white vertical stimuli at 1 Hz (2 reversals per second), and 50 candela/m^2^ delivered by the pattern stimulator of a Diagnosys Celeris system (Diagnosys LLC, Lowell, MA, USA). Then, 300 traces were recorded per eye, and averaged waveforms were calculated in which amplitudes (µV) were measured from the P1 peak to the N2 trough. All pERG measurements were recorded while maintaining constant body temperature between 37 °C and 38 °C using the system’s heat pads. 

### 4.5. Analysis of RGC Survival

To analyze RGC density, eyes were fixed in 4% paraformaldehyde for two hours and rinsed in PBS. Retinas were dissected and permeabilized in 0.3% Triton-X100/PBS for 6 h followed by three freeze and thaw cycles and blocking in 1%BSA/0.3% Triton-X100/PBS for 1 h at room temperature. Retinas were incubated with goat-anti Brn3a primary antibody [80] (Santa Cruz, TX, diluted 1:200 in 1%BSA/0.3% Triton-X100/1% DMSO/PBS) at 4 °C for 48 h on a rocker platform. After washes in PBS, binding was visualized following incubation in a donkey anti-goat Alexa Fluor 546 secondary antibody solution (Invitrogen, Carlsbad, CA, USA, 1:200 in PBS) for 3 h at room temperature in the dark. After extensive washing in PBS, retinas were wholemounted using Vectashield (Vector Laboratories, Burlingame, CA, USA), and eight images from each retina were taken at predetermined locations peripherally, mid-peripherally, and centrally at 20× magnification using an Olympus BX41 microscope (Olympus, Center Valley, PA, USA). These 24 images represent 23.8% of the total mouse retinal area [81]. Brn3a^+^ cells were counted manually by a masked observer using the cell counter plugin for ImageJ software (National Institutes of Health, Bethesda, MD, USA). RGC density per mm^2^ was calculated by averaging the 8 values per area of each retina. 

### 4.6. RNA-Sequencing

Elevated IOP was induced in the eyes of C57BL/6 mice as described above. Three weeks after elevation of IOP, eyes were harvested, and total RNA was extracted. Four pools of RNA, each containing material from two eyes obtained from two distinct animals, were generated from eyes with elevated IOP as well as 4 pools from naïve mice. Each pool was converted to a sequencing library and sequenced on a HiSeq4000 sequencing system (Illumina, San Diego, CA, USA) generating, on average, 19.4 million reads for each pool. Reads were aligned to the mm10 reference genome using STAR aligner software [82], and gene and transcript level expression values in length normalized transcripts per million were generated using the R package FeatureCounts. Differential gene expression analysis was carried out using DEseq2 [83], and the resulting data were further analyzed using Ingenuity Pathway analysis and String (https://string-db.org/; accessed on 18 May 2018) [84]. Cluster analysis was performed by String DB software using gene lists of significantly up-regulated or down-regulated genes with a cut-off *p*-value > 0.05 and a high confidence interaction score setting of 0.7.

### 4.7. Immunohistology

To analyze structural changes to the retina, eyes from animals with three weeks IOP elevation post Ad5.MYOC injection and from control mice were fixed in 4% paraformaldehyde for two hours and rinsed in PBS. After cryopreservation using sucrose embedding, sagittal 7 µm sections were washed three times for 5 min in PBS and blocked in 1%BSA/PBS for 1 h at room temperature. Sections were incubated with rat-anti C1q (Abcam, Cambridge, UK, diluted 1:50 in 1%BSA/PBS), goat-anti Brn3a (Santa Cruz, Dallas, TX, USA, diluted 1:200 in 1%BSA/PBS), goat-anti PSD95 (Abcam, diluted 1:1000 in 1%BSA/PBS), or rabbit-anti GABA_A_ Receptor A1 (Millipore Sigma, Burlington, MA, USA, 1:500 in 1%BSA/PBS) at 4 °C overnight. Then, slides were washed and incubated for three hours at room temperature in goat anti-rat Alexa Fluor 488 (Invitrogen, Carlsbad, CA, USA, 1:500 in PBS) donkey anti-goat Alexa Fluor 546 (Invitrogen, 1:500 in PBS) or donkey anti-rabbit Alexa Fluor 488 (Invitrogen, 1:500 in PBS). Slides were washed three times for 5 min in PBS followed by a rinse in 70% EtOH and distilled water. After coverslipping using Aquamount, dry slides were imaged on a Zeiss 710 confocal microscope (Carl Zeiss, Thornwood, NY, USA). 

### 4.8. Statistics

IOP and RGC data are given as mean ± standard deviation (SD). For statistics including more than two groups, *p*-values are calculated using ANOVA followed by Turkey’s post hoc test. RNA seq. data are displayed as mean values of fragments per kilobase of transcript per million mapped reads (FPKM) ± SD. *p*-values between control retinas and retinas three weeks after Ad5.MYOC-induced ocular hypertension were calculated using Student’s *t*-test. All calculations were performed using GraphPad Prism v7.04 (GraphPad Software, San Diego, CA, USA), and *p*-values < 0.05 were considered statistically significant.

## 5. Conclusions

The intraocular administration of Ad5 viral construct expressing a pathogenic variant of myocilin leads to robust and chronic IOP elevation in mice and results in a slow-progressive RGC loss. Early retinal changes in hypertensive eyes feature key characteristics of glaucomatous degeneration including glial activation, inceptive neuroinflammation, activation of the complement system, and synaptic loss. These molecular changes are accompanied by functional impairment i.e., a significant decline of the pERG amplitude before RGC loss becomes apparent. Furthermore, our RNA sequencing data with subsequent pathway analysis indicate increased antigen presentation in conjunction with HSP-mediated proteolysis as a response to activation of the ubiquitin–proteasome system. This observation merits further investigation and may provide a possible mechanism as to how autoimmune responses in glaucoma are initiated.

## Figures and Tables

**Table 1:** Gene expression changes after three weeks of Ad5.MYOC-induced ocular hypertension (Ad5.MYOC) when compared to naïve age-matched controls.

**Downregulated mRNA Expression**				
	**Gene**	**WT (FPKM ± SD)**	**Glaucoma (FPKM ± SD)**	***t* Test**
GABA_A_ receptor and GABA neurotransmitter recycling	*Adcy2*	1871 ± 81	1671 ± 82	0.0133
	*Cacna1d*	979 ± 32	874 ± 57	0.0182
	*Gabbr2*	1790 ± 53	1543 ± 87	0.0029
	*Gabra1*	2367 ± 121	2034 ± 182	0.0226
	*Gabrb2*	628 ± 29	565 ± 36	0.0328
	*Gabrb3*	2188 ± 113	1954 ± 110	0.0249
	*Gabrg3*	300 ± 11	268 ± 9	0.0037
	*Gad1*	6470 ± 379	5441 ± 397	0.0095
	*Gad2*	1269 ± 68	1113 ± 76	0.0226
	*Glul*	47,282 ± 621	33,233 ± 4840	0.0012
	*Gnao1*	8319 ± 338	7421 ± 507	0.0257
	*Gng2*	1226 ± 42	1066 ± 82	0.0132
	*Slc6a1*	10,796 ± 661	9056 ± 913	0.0214
	*Slc38a1*	5525 ± 205	4845 ± 405	0.0244
Cholinergic and glutamatergic synapses	*Adcy2*	1871 ± 81	1671 ± 82	0.0133
	*Cacna1d*	979 ± 32	874 ± 57	0.0182
	*Camk4*	566 ± 33	502 ± 23	0.0183
	*Chrm2*	195 ± 3	172 ± 6	0.0004
	*Chrna4*	624 ± 34	556 ± 34	0.0290
	*Dlgap3*	1616 ± 87	1449 ± 43	0.0166
	*Glul*	47,282 ± 621	33,233 ± 4840	0.0012
	*Gnao1*	8319 ± 338	7421 ± 507	0.0257
	*Gng2*	1226 ± 42	1066 ± 82	0.0132
	*Gria2*	1711 ± 56	1469 ± 70	0.0016
	*Grm2*	339 ± 12	299 ± 24	0.0284
	*Grm6*	2334 ± 163	2005 ± 100	0.0136
	*Kcnj2*	217 ± 3	195 ± 7	0.0015
	*Prkca*	6764 ± 127	5909 ± 350	0.0037
	*Shank1*	1449 ± 95	1263 ± 67	0.0186
	*Slc5a7*	311 ± 17	268 ± 10	0.0054
	*Slc38a1*	5525 ± 205	4845 ± 406	0.0244
Axon guidance	*Dpysl2*	1726 ± 56	1518 ± 76	0.0046
	*Dpysl5*	1231 ± 24	1094 ± 56	0.0042
	*Efnb3*	522 ± 39	451 ± 32	0.0327
	*Epha8*	1215 ± 71	997 ± 64	0.0038
	*L1cam*	1877 ± 119	1655 ± 83	0.0223
	*Plxna1*	1114 ± 63	998 ± 47	0.0254
	*Rgs3*	726 ± 38	646 ± 38	0.0251
	*Robo2*	1986 ± 112	1742 ± 139	0.0334
	*Sema4g*	1067 ± 46	905 ± 80	0.0123
	*Sema6b*	791 ± 37	708 ± 28	0.0115
	*Slit1*	523 ± 24	468 ± 18	0.0109
	*Slit2*	2512 ± 179	2146 ± 138	0.0176
	*Srgap3*	4500 ± 193	3953 ± 276	0.0176
	*Unc5b*	338 ± 12	292 ± 28	0.0228
**Upregulated mRNA Expression**				
	**Gene**	**WT (FPKM ± SD)**	**Glaucoma (FPKM ± SD)**	***t* Test**
Antigen processing and presentation	*Ctss*	561 ± 207	1319 ± 354	0.0101
	*H2-T23*	248 ± 48	452 ± 141	0.0336
	*Ifi30*	151 ± 12	201 ± 29	0.0184
	*Psme2*	500 ± 19	600 ± 49	0.0090
	*Tapbp*	1709 ± 136	2287 ± 268	0.0085
Complement cascade	*A2m*	476 ± 157	1269 ± 297	0.0033
	*C1qa*	297 ± 92	656 ± 154	0.0072
	*C1qb*	324 ± 116	715 ± 179	0.0107
	*C1qc*	295 ± 102	589 ± 133	0.0125
	*C3ar1*	52 ± 4	65 ± 6	0.0109
	*C4b*	835 ± 599	2781 ± 1120	0.0221
	*Cfh*	309 ± 44	412 ± 54	0.0248
	*Cfi*	68 ± 2	83 ± 9	0.0199
	*Pros1*	359 ± 41	513 ± 111	0.0405
	*Serping1*	411 ± 186	1081 ± 489	0.0430
Cytokine–cytokine receptor signaling	*Ccr5*	44 ± 3	53 ± 4	0.0117
	*Csf1r*	444 ± 112	865 ± 175	0.0066
	*Cx3cr1*	199 ± 34	306 ± 54	0.0153
	*Cxcl10*	41 ± 2	53 ± 9	0.0416
	*Dock2*	61 ± 4	76 ± 8	0.0194
	*Gnb3*	8209 ± 663	11,825 ± 1307	0.0026
	*Hck*	41 ± 2	50 ± 5	0.0225
	*Il10ra*	62 ± 3	75 ± 6	0.0066
	*Jak3*	138 ± 13	206 ± 40	0.0175
	*Ltbr*	198 ± 18	252 ± 38	0.0430
	*Ncf1*	65 ± 5	81 ± 9	0.0233
	*Osmr*	269 ± 55	507 ± 129	0.0146
	*Stat1*	1613 ± 293	2730 ± 770	0.0351
	*Stat2*	1117 ± 88	1343 ± 158	0.0467
	*Stat3*	1815 ± 280	3209 ± 846	0.0204
	*Tnfrsf1a*	308 ± 60	517 ± 139	0.0334
	*Vav1*	50 ± 3	61 ± 6	0.0173
Clusters of differentiation	*Cd52*	57 ± 5	74 ± 9	0.0177
	*Cd53*	63 ± 3	76 ± 9	0.0296
	*Cd63*	975 ± 69	1351 ± 223	0.0183
	*Cd68*	83 ± 9	108 ± 16	0.0323
	*Cd72*	46 ± 4	55 ± 4	0.0171
Ribosomal subunits	*Rpl10*	2480 ± 74	3010 ± 250	0.0067
	*Rpl13a*	7349 ± 247	8843 ± 789	0.0112
	*Rpl17*	3028 ± 134	3756 ± 362	0.0092
	*Rpl19*	2983 ± 133	3615 ± 268	0.0055
	*Rpl32*	2190 ± 69	2631 ± 190	0.0048
	*Rpl41*	2805 ± 98	3427 ± 337	0.0121
	*Rpl9*	7008 ± 340	8670 ± 961	0.0172
	*Rplp0*	4190 ± 138	5225 ± 478	0.0059
	*Rplp1*	5660 ± 98	6845 ± 558	0.0058
	*Rps10*	2794 ± 87	3379 ± 284	0.0077
	*Rps12*	2473 ± 105	2973 ± 223	0.0066
	*Rps14*	4689 ± 218	6181 ± 786	0.0106
	*Rps15*	5240 ± 146	6408 ± 623	0.0107
	*Rps18*	3420 ± 135	4242 ± 379	0.0065
	*Rps19*	2340 ± 113	2826 ± 228	0.0088
	*Rps20*	2210 ± 58	2656 ± 208	0.0061
	*Rps24*	3900 ± 105	4802 ± 366	0.0032
	*Rps25*	3129 ± 138	3762 ± 316	0.0105
	*Rps27a*	3820 ± 118	4650 ± 348	0.0040
	*Rps3*	6347 ± 170	7880 ± 842	0.0118
	*Rps9*	3916 ± 142	4702 ± 311	0.0037
	*Rras*	225 ± 16	299 ± 53	0.0373
	*Rtp4*	63 ± 4	80 ± 11	0.0207

## Data Availability

RNA-sequencing data will be made available upon request. Please contact the corresponding author.
